# Survey of calcium supplementation to prevent preeclampsia: the gap between evidence and practice in Brazil

**DOI:** 10.1186/1471-2393-13-206

**Published:** 2013-11-11

**Authors:** Erika Barbosa Camargo, Luci Fabiane Scheffer Moraes, Celsa Moura Souza, Rita Akutsu, Jorge Maia Barreto, Edina Mariko Koga da Silva, Ana Pilar Betrán, Maria Regina Torloni

**Affiliations:** 1 Internal Medicine Department, São Paulo Federal University (UNIFESP), São Paulo, Brazil; 2 Physical Activity and Health Research Laboratory, South Santa Catarina University (UNISUL), Tubarão, Santa Catarina, Brazil; 3 Public Health Department, Amazonas Federal University (UFAM), Manaus, Amazonas, Brazil; 4 Nutrition Department, Brasília Federal University (UnB), Brasília, Brazil; 5 Federal University of Piauí, Teresina, Piauí, Brazil; 6 Department of Reproductive Health and Research, World Health Organization, Geneva, Switzerland

**Keywords:** Preeclampsia, Eclampsia, Calcium, Dietary supplements, Primary prevention, Calcium dietary, Developing countries

## Abstract

**Background:**

Preeclampsia is a major cause of maternal and perinatal morbidity and mortality worldwide and especially in Latin America. High quality evidence indicates that calcium supplementation during pregnancy significantly reduces the incidence of preeclampsia and its consequences, including severe maternal morbidity and death. Few studies have assessed the implementation of this intervention in clinical practice. The study aimed to assess the proportion of pregnant women who received calcium supplements in Brazilian public antenatal care clinics.

**Methods:**

This cross-sectional study interviewed women waiting for antenatal care visits in 9 public clinics in 4 Brazilian cities in 2010-2012. Trained interviewers used a standardized anonymous questionnaire to collect socio demographic and obstetric data, information on ingestion of dairy products and on prescriptions received during current pregnancy.

**Results:**

A total of 788 valid questionnaires were analyzed. Participants were young (mean age 25.9), mostly multiparous (71.3%) and in the 2^nd^ or 3^rd^ trimesters of pregnancy at the time of interview (87.6%). Only 5.1% (40/788) had received a prescription for calcium supplements. Based on their reported ingestion of dairy products, the mean daily dietary calcium intake of the participants was 210 (+ 265) mg/day and over 90% consumed less than 1 g of calcium/day.

**Conclusions:**

Despite good quality evidence indicating the benefits of this practice especially for women with low calcium diets, less than 6% of a sample of women receiving antenatal care in Brazilian public clinics received a prescription for calcium supplements. There is an urgent need to upscale the implementation of this life-saving intervention.

## Background

Each year, almost 60,000 women die in the world due to hypertensive disorders of pregnancy [1]. Hypertension is the leading cause of maternal mortality (MM) in Latin America [2,3]. According to the latest national estimates, pre-eclampsia (PE) and eclampsia (E) are the single largest contributors to the MM rate of Brazil, accounting for approximately 20% of all maternal deaths in that country [4]. Hypertensive disorders are also a major cause of elective preterm births as well as perinatal morbidity and mortality [5-7]. Each year, the Brazilian public health system spends over $14 million US dollars in direct costs for the treatment of PE/E, a substantial expenditure for a developing country [8].

PE, defined as hypertension and proteinuria with onset in the 2^nd^ half of pregnancy [9], affects 6-10% of all pregnant women and 2-8% of them will develop severe forms of the disease including eclampsia, characterized by the appearance of seizures [10]. Although the pathogenesis of PE is not yet completely understood, existing evidence indicates that the main initiating events are placental hypoperfusion, increased production of inflammatory and anti-angiogenic mediators, followed by systemic endothelial damage, disseminated vasospasm, generalized tissue hypoperfusion and aggravation of placental ischemia, thus perpetuating the vicious cycle [11-14]. Currently there is no effective treatment for PE/E and delivery is the only option to halt the disease process [15].

Over 30 years ago, observational studies identified an inverse relationship between maternal calcium (Ca) intake and the incidence of PE [16,17]. Based on these findings, a series of randomized controlled trials (RCT) were carried out to evaluate the effectiveness of Ca supplementation in the prevention of PE [18-20]. Over the last 15 years, several systematic reviews pooled the results of these trials, showing the benefits of this intervention [21-24]. According to a systematic review of individual data from 12 RCTs which included over 15,000 participants, Ca supplementation starting in the 2^nd^ trimester of pregnancy reduces the risk of developing PE by 64% and the risk of MM or severe maternal morbidity by 20%, in women with low dietary Ca intake, defined as less than 900 mg/day [25]. In 2011 the World Health Organization (WHO) recognized Ca supplementation during pregnancy as an effective intervention to prevent PE and to reduce MM and recommended that women with low dietary intake or at high risk for PE should receive 1.5-2.0 g of Ca daily, starting in the 2^nd^ trimester [26].

PE is associated with a large burden of morbidity and mortality in low-resource settings, especially in Latin America, and there is a body of high quality evidence indicating the potential benefits of Ca supplementation in pregnancy; yet the actual implementation of this intervention into clinical practice has not been extensively investigated. In 2010 Silva et al. interviewed 250 high-risk pregnant or post-partum Brazilian women and reported that only 10% of them had received prescriptions for Ca supplements during pregnancy [27]. We identified no other publications assessing the antenatal prescription of Ca to prevent PE. Our main objective was to assess the proportion of women who received a prescription of Ca supplements during pregnancy in a sample of Brazilian public healthcare clinics.

## Methods

This cross-sectional study interviewed women receiving antenatal care in the public Brazilian healthcare system. The interviews were performed between October 2010 and June 2012 in nine public healthcare clinics located in four cities in different geographic regions of Brazil: Coari (Amazonas state, north region, 3 clinics), Brasília (Federal District, central region, 1 clinic), Piripiri (Piauí state, central region, 3 clinics) and Tubarão (Santa Catarina state, southern region, 2 clinics).These clinics offered free antenatal care to the general obstetric population, i.e. mostly low and medium risk pregnant women. The Brazilian national health system offers free antenatal care to all women in the country and recommends the use of folic acid and iron supplements to all pregnant women. There is no national guideline on other supplements during pregnancy. Calcium carbonate tablets containing 500 mg of elemental Ca are distributed free of charge by the national health system and dispensed locally in any public healthcare center, to all patients who have a medical prescription for this supplement.

Pregnant women carrying a live fetus, of any parity and gestational age, booked for antenatal care at one of the selected centers, and capable of answering an oral questionnaire were considered eligible to participate. Women waiting for their 1^st^ antenatal care visit were excluded. Interviewers approached the women in the waiting rooms of the health facilities during morning periods, explained the objectives of the study and invited them to participate. Participants who fulfilled the selection criteria and agreed to participate were asked to sign an informed consent form and then interviewed using a standardized oral questionnaire. The questionnaire was designed and tested by the authors and consisted of close and open-ended questions to collect information on socio demographic characteristics, obstetric history, current pregnancy, usual dairy intake and medical prescriptions received so far, including Ca supplementation. The question on dairy consumption asked participants what was their current usual intake of milk, cheese or other milk products. The Brazilian food composition table [28] was used to estimate the amount of dietary Ca ingested by the participants based on their consumption of dairy products. Dairy intake was converted to estimated Ca intake based on the reported ingestion of milk (one 200 ml cup =246 mg Ca) and cheese (one 30 g slice = 282 mg Ca). To assess the adequacy of Ca intake, the latest recommendations of the Dietary Reference Intakes - DRI were used [29,30]. According to these recommendations, adult pregnant women should have a daily Ca ingestion of 1,000 mg and pregnant adolescents (14-18 years) should have a daily ingestion of 1,300 mg. Pregnant women with an average Ca intake < 900 mg/day are classified as having a low dietary intake of Ca [25].

The interviews were anonymous, lasted an average of 10 minutes and were conducted individually and privately. Participants were encouraged to check their antenatal care booklet, which was in their hands at the time of the interview, to confirm information given to the interviewer. Women were informed that they could decline to answer any question. Questionnaires with more than five unanswered questions were excluded from the analyses.

The interviewers were three medical students and nine nutrition undergraduates who were specifically trained for this study. The interviewers collected data daily during an average of five weeks in each of the participating clinics.

Sample size was calculated using the frequency of Ca prescription reported by Silva 2010 [27] of 10%. With an absolute error of 2.5% and a significance level of 5%, a sample of 553 participants was calculated. Considering possible losses, the planned sample was 800 participants. The cities and public healthcare clinics included in the study were selected as a convenience sample.

The completed questionnaires were sent by mail to the first author who entered the data into an electronic database. Results are presented descriptively as frequencies and percentages for categorical variables, and as means and standard deviation (SD) for continuous variables. Relevant outcomes are presented with their 95% confidence intervals (CI).

Participants were divided in two subgroups according to their risk for PE, based on widely accepted parameters [31-34]. Participants with any of the following characteristics were considered at high risk for PE: nulliparas, women <20 or >35 years, diabetics (type 1 or 2) or women with a history of hypertension in a previous pregnancy. All other participants, i.e. those without any of the previous characteristics, were classified as being at low risk for PE. We analyzed possible differences in the rate of Ca prescription between these two groups using the Chi square test. P values < 0.05 were considered significant. The software SPSS version 1.6 (IBM^®^ SPSS^®^, Chicago, U.S.A) was used for analyses.

The study was approved by São Paulo Federal University’s ethics committee and all participants provided free informed consent. The study complies with the ethical principles of the Helsinki declaration.

## Results

A total of 832 eligible women were invited to participate and 32 declined. Of the 800 who agreed to answer the questionnaire, 12 refused to answer five or more questions and were excluded from the study, resulting in 788 valid questionnaires included in the final analyses. Table 1 presents the main characteristics of the participants. Age ranged from 13 to 42 years and 17.6% were adolescents. Most participants were living with a partner (82.1%), multiparous (71.3%) and reported that the current pregnancy had not been planned (56%). Nearly 90% of participants were interviewed in the 2^nd^ or 3^rd^ trimesters of pregnancy and almost half of them had attended at least five antenatal visits at the time of the interview. A total of 51 women (6.5%) smoked and 61 (7.7%) consumed alcohol at least once a week. A total of 250 (31.7%) women were classified as being at high risk for PE, the main factor was nulliparity.

**Table 1:** Main characteristics and dietary calcium intake of 788 Brazilian pregnant women

**Variable**	**Values**^**1**^	
**Age, years**	25.9	(6.3)
**Marital status**		
Married or living with partner	647	(82.1)
Single	127	(16.1)
Separated/divorced	14	(1.8)
**Parity**		
0	226	(28.7)
1	245	(31.1)
2 or +	317	(40.2)
**Unplanned pregnancy**	440	(55.8)
**Gestational age at the time of the interview**		
Mean (SD), weeks	24.3	(8.3)
1^st ^trimester	98	(12.4)
2^nd ^trimester	315	(40.0)
3^rd ^trimester	375	(47.6)
**Number of antenatal care visits up to the moment**		
1	112	(14.2)
2 to 4	314	(39.9)
5 or +	362	(45.9)
Women at high risk for PE^*^	250	(31.7)
**Current milk intake (cups/day)**		
0	189	(24.0)
1 (246 mg)^2^	282	(35.8)
2 - 3 (492 mg – 738 mg)	267	(33.9)
4 or + (984 mg or +)	50	(6.3)
**Current cheese intake**		
Up to 1 portion^3^/day	130	(16.5)
Up to 1 portion/week	199	(25.3)
Up to 1 portion/month	50	(6.3)
Rarely^4 ^or never	409	(51.9)

*Any of the following: nulliparity, age <20 or >35 years, diabetes mellitus or history of hypertension in a previous pregnancy.

^1^All values expressed as mean (SD) or N (%).

^2^Equivalent calcium value.

^3^One portion is 30 g of cheese.

^4^Less than one portion per month.

Approximately 60% of the women reported that they drank only up to one cup of milk (246 mg Ca) daily, while only 6.3% drank four or more cups of milk daily (95% CI 4.8% - 8.2%). Over half of the participants never or rarely (less than once a month) ate cheese. The ingestion of other dairy products (including yogurt, sour cream or cream spreads) was even less frequent, with 79% (622) of the women reporting that these were consumed less than once a month. Only 3% (4/139) of the adolescents included in the survey drank four or more cups of milk per day (984 mg Ca) and 58.2% reported that they rarely or never ate cheese. Based on their reported ingestion of dairy products, the mean daily dietary Ca intake of the 788 participants was 210 (± 265) mg and over 90% consumed less than 1 g of Ca/day. The mean daily dietary Ca intake from dairy products reported by adolescents was 313 (± 262) mg.

Less than 6% (N = 40) of the participants (95% CI 3.7% - 6.8%) reported that they had received a prescription for Ca supplements during their current pregnancy (Table 2). There were no significant differences in the women at high versus low risk for PE (5.2% versus 5.0%, respectively). Most (60%) of the 40 women who had received a Ca prescription did not know the reasons for this supplementation; only three reported to have been told that these tablets were to prevent problems related to high blood pressure. Approximately one third of the participants (N = 252) had been advised by their physician to increase the ingestion of dairy products during pregnancy. Over half (144/252) of these women received instructions to drink a total of 1-2 glasses of milk/day, while approximately 40% (98/252) informed that their doctor had not been clear about the type and quantity of dairy product that they should consume daily (Table 2). Overall, less than 1% of the participants (4/788) were encouraged to consume enough dairy products to attain international DRIs for pregnant women.

**Table 2:** Calcium prescription and advice on the ingestion of dairy products received by 788 Brazilian pregnant women

	**Values**	
	**N**	**(%)**
**Did physician prescribe calcium?**		
Yes	40	(5.1)
No	694	(88.1)
Unsure/Doesn’t know	54	(6.8)
**Did physician explain the reason for calcium prescription**^**2**^**?**		
Yes	16	(40.0)
No	24	(60.0)
**Reason for calcium prescription**^**2**^**:**		
For bones (baby or mother)	10	(62.5)
For blood pressure	3	(18.8)
Calcium deficiency	2	(12.5)
Anemia	1	(6.3)
**Did physician tell you to eat more dairy products?**		
Yes	252	(32.0)
No	536	(68.0)
**Recommendations given by physician**^**2**^**:**		
Drink 1–2 cups of milk/day	144	(57.1)
Drink 3–4 cups of milk/day	10	(4.0)
Unclear/did not specify	98	(38.9)

^2^Over total answering Yes to previous question.

## Discussion

Less than 6% of almost 800 women receiving antenatal care in nine public Brazilian healthcare clinics were given a prescription of Ca supplements during pregnancy and most of them did not know why they were taking it. Additionally, based on their reported dairy product ingestion, over 90% of the participants consumed less than 1 g Ca/day. Less than one third of the women informed that their physicians had told them to increase the ingestion of these products during pregnancy and when they did, in over 95% of the cases, the recommendations did not comply with minimum DRIs for pregnancy.

Although our main objective was not to estimate dietary intake of Ca by pregnant women, the participants’ average dietary Ca intake from dairy products was less than 400 mg/day. Given that milk products were the only food items included in the questionnaire, the actual daily Ca intake of these women was probably higher than this, although it is unlikely that it would be close to the 1 g/day recommended for pregnancy. According to the latest national food inquiry survey, the mean dietary Ca intake of adult Brazilian women (19-59 years) is only 438 mg/day and 90.7% of them do not reach the recommended dietary calcium intake for their age [35]. According to this same survey, dairy products are the main source of dietary Ca for Brazilian adults. Only a few, small studies have assessed the Ca ingestion of Brazilian pregnant women and they focused on specific groups, such as overweight and adolescent participants. According to these studies, mean daily Ca intakes ranged from 586.6 mg to 842.9 mg/day [36-38]. This low dietary Ca intake is typical of diets in Latin America and Asia [39-41] and contrasts with the daily 1200 mg Ca/day of American [42] and European pregnant women [43,44], with some exceptions [45].

The only other study that evaluated Ca prescription to prevent PE was also carried out in Brazil and reported that 10% of 250 women at high risk for PE had received Ca supplements during pregnancy [27]. The somewhat higher proportion of women receiving Ca supplementation in that study could be attributed to the fact that those investigators interviewed exclusively high-risk women managed in tertiary teaching university hospitals.

This study had several strong points. To the best of our knowledge, it is the largest study to analyze Ca supplementation during pregnancy to prevent PE. In addition, data collection followed rigorous methodology using a standardized anonymous questionnaire and trained interviewers. However, we acknowledge that the assessment of dietary Ca could have been more complete if we had collected information on the ingestion of other non-dairy sources of Ca. A limitation of this study was the use of a convenience sample. However, the main characteristics of our participants are similar to the general population of Brazilian reproductive age women reported in a recent national survey on maternal and child health [46]. Nevertheless, the results of this survey cannot be generalized to other settings or countries. Although our sample most likely over-represented urban women, it would be expected that the dietary Ca intake of pregnant rural women would be even lower, as suggested by the latest national inquiry on food habits [35]. Finally, as with any other patient survey, the data analyzed were based exclusively on the answers given by the participants and are therefore subject to recall bias [47] and do not necessarily reflect the exact practice of the physicians. Future studies should interview health professionals involved in prenatal care, to assess their knowledge, attitude and practice regarding Ca supplementation during pregnancy, in order to identify the main facilitators and barriers to upscale the implementation of this intervention.

Our findings indicate that Brazilian doctors working in the public health system do not routinely prescribe Ca to their pregnant patients, despite the free availability of these supplements in the public health system. This is possibly due to their lack of knowledge about the benefits of Ca supplementation to prevent PE. Besides the need for continued medical education of these professionals, the guidelines currently used in the country’s public health care system need to be reviewed and updated, to encourage the use of this, as well as other evidence-based interventions for maternal health. Besides the evident benefits of Ca supplementation for the individual patients and their families, decreasing the number of cases of PE/E can also have significant cost saving implications for the national public health system.

Most of the research to date has focused on supplementation starting in the 2^nd^ trimester of pregnancy and although current evidence supports Ca supplementation during pregnancy for the prevention of PE, it has been hypothesized that the effect could be even more dramatic if supplementation started earlier. WHO is currently coordinating a multi-country randomized trial to assess if periconceptional Ca supplementation reduces the incidence of recurrent PE more effectively than supplementation starting in the 2^nd^ trimester [48]. Results of this trial may increase the value of Ca supplementation in the future.

Despite existing WHO evidence-based recommendations indicating that Ca supplementation in pregnancy is beneficial for women with low dietary Ca intake, our study indicates that this intervention is not being used in the daily practice of public healthcare clinics in Brazil. This finding is not unexpected since the gap between good evidence and implementation is common in the history of obstetric practice. For example, several decades passed between the publication of systematic reviews pointing that antenatal corticosteroids significantly reduced mortality and morbidity of premature babies, before this intervention was widely implemented worldwide [49]. Similarly, it took almost a century, during which thousands of unnecessary maternal deaths occurred, until magnesium sulfate became widely used for the prevention and treatment of eclampsia [50].

Implementation of evidence-based obstetrical practice remains a challenge. Time, as well as political determination, strategic efforts, financial and human resources are necessary to transform good evidence into good clinical practice. Investments in implementation research are also needed to develop strategies that will help to reduce the still ubiquitous large gap between evidence and practice.

## Conclusions

Ca supplementation is given to less than 6% of women receiving antenatal care in a sample of public clinics in Brazil. Strategic efforts are needed to enhance the implementation of this evidence-based intervention. Upscaling this practice could lead to a significant reduction in the incidence of PE/E, reducing associated maternal and perinatal morbidity and mortality as well as costs for the Brazilian public health system.

## Abbreviations

Ca: Calcium; CI: Confidence interval; DRI: Daily recommended intakes; E: Eclampsia; PE: Preeclampsia; RCT: Randomized clinical trial; SD: Standard deviation; WHO: World Health Organization.

## Competing interests

All authors declare that they have no competing interests.

## Authors’ contributions

EBC designed the study, supervised data collection, performed analyses and interpretations and drafted the manuscript. LFSM, CMS, RA and JB supervised data collection, participated in data analyses and interpretation. EMKS conceived and designed the study, performed analyses and was involved in revising the manuscript critically for important intellectual content. APB contributed to interpretation and analyses of data and was involved in revising the manuscript critically for important intellectual content. MRT performed analyses and interpretations and drafted the manuscript. All authors read and approved the final manuscript.

## Pre-publication history

The pre-publication history for this paper can be accessed here:

http://www.biomedcentral.com/1471-2393/13/206/prepub
